# Safety and Clinical Effects of Switching From Intravenous to Oral Nimodipine Administration in Aneurysmal Subarachnoid Hemorrhage

**DOI:** 10.3389/fneur.2021.748413

**Published:** 2021-11-16

**Authors:** Jennifer Göttsche, Nils Schweingruber, Julian Christopher Groth, Christian Gerloff, Manfred Westphal, Patrick Czorlich

**Affiliations:** ^1^Department of Neurosurgery, Hamburg University Medical Center, Hamburg, Germany; ^2^Department of Neurology, Hamburg University Medical Center, Hamburg, Germany

**Keywords:** subarachnoid hemorrhage, Transcranial Doppler, delayed cerebral ischemia, vasospasm, nimodipine, norepinephrine, catecholamine

## Abstract

**Objective:** Several guidelines recommend oral administration of nimodipine as vasospasm prophylaxis after aneurysmal subarachnoid hemorrhage (SAH). However, in clinical practice, the drug is administered orally and intravenously (i.v.), depending on clinical conditions and local treatment regimens. We have therefore investigated the safety and clinical effects of switching from i.v. to oral nimodipine therapy.

**Methods:** Patients with aneurysmal SAH between January 2014 and April 2018 and initial i.v. nimodipine therapy, which was subsequently switched to oral administration, were included in this retrospective study. Transcranial Doppler sonography (TCD) of the vessels of the anterior circulation was performed daily. The occurrence of vasospasm and infarction during the overall course of the treatment was recorded. Statistical level of significance was set to *p* < 0.05.

**Results:** A total of 133 patients (mean age 55.8 years, 65% female) initially received nimodipine i.v. after aneurysmal SAH, which was subsequently switched to oral administration after a mean of 12 days. There were no significant increases in mean flow velocities on TCD after the switch from i.v. to oral nimodipine administration regarding the anterior cerebral artery. For the middle cerebral artery, an increase from 62.36 to 71.78 cm/sec could only be detected in the subgroup of patients with infarction. There was no clustering of complicating events such as new-onset vasospasm or infarction during or after the switch.

**Conclusions:** Our results do not point to any safety concerns when switching nimodipine from initial i.v. to oral administration. Switching was neither associated with clinically relevant increases in TCD velocities nor other relevant adverse events.

## Introduction

Cerebral vasospasm (CVS) and delayed cerebral ischemia (DCI) remain common and severe complications after aneurysmal subarachnoid hemorrhage (SAH) and are jointly responsible for the high morbidity and mortality, which is still above 20% in recent publications (1–3). About 30% of all patients develop DCI in the course of SAH (4). The underlying pathophysiology is thought to be of multifactorial origin: In addition to angiographic vasospasm, cortical spreading depolarization, microthrombosis, microcirculatory dysfunction and neuro-inflammation have been investigated recently as factors causing DCI (5–9).

Due to sedation, repetitive neurological exams cannot be performed to assess clinical deterioration, therefore Transcranial Doppler sonography (TCD) can be used as one of several methods to obtain indication of vasospasms especially in large intracranial arteries (10–14).

A treatment of SAH patients with nimodipine was shown in several studies to be effective in reducing incidence of poor outcome and severe neurological deficits after aneurysmal SAH but showed no influence on the occurrence of CVS or DCI (15–18). Although the underlying neuroprotective mechanism of nimodipine is yet not fully understood a positive effect on the functional outcome of SAH patients has been confirmed (19, 20). Administration of nimodipine is a well-established treatment modality and can happen orally or intravenously (i.v.) (21).

Based on the strong study evidence and current guideline recommendations, nimodipine should be given p.o. whenever possible (15–18, 22). However, in the acute phase, many patients cannot take the oral medication. There are considerations as to whether oral administration in analgosedated patients could result in lower bioavailability compared to i.v. administration (23, 24). It has therefore become clinical practice to administer the drug intravenously in the acute phase.

In a recent retrospective analysis <50% of all patients on oral nimodipine therapy received the full dose as a consequence of its blood pressure lowering effect, which should be avoided to prevent cerebral infarctions (25). This side effect of nimodipine counteracts the desired induced hypertension, forcing an intermittent interruption, lowering dose of application or concomitant intravenous administration of norepinephrine is often necessary.

The intravenous administration of nimodipine therefore represents an alternative and has been investigated in several studies in the past due to its relevance (26–28). The aim of this analysis was to systematically evaluate our clinical observations of whether neurological and intensive care parameters change immediately due to the change from i.v. to oral administration in the course of the disease with a special focus on the measured blood flow velocity in TCD and the dosage of norepinephrine. If significant differences were to be found, this could be a basis for further investigations, since randomized data are not available at present.

The aim of this analysis was to evaluate our clinical observations of whether neurological and intensive care parameters change directly as a result of switching from i.v. to oral administration in the course of disease, focusing on measured blood flow velocity in TCD and norepinephrine dosing.

## Materials and Methods

### Study Population

Only patients admitted with aneurysmal SAH between January 2014 and April 2018 and initial i.v. nimodipine therapy over at least 48 h, which was subsequently switched to oral administration, were included in this retrospective single center study, resulting in 133 of 299 SAH patients available for further analysis (for details see Supplementary Figure 1). Basic clinical characteristics of our study population including clinical parameters and SAH-relevant events on intensive care unit (ICU) at admission and during hospitalization as well as medical history were collected. Data collection included demographic information, aneurysm location, information on antiplatelet aggregation medication, pre-existing conditions and distinct clinical evaluation scores (Glasgow Coma Scale, Hunt & Hess grading system, WFNS grading system, Fisher score). The drug doses were extracted from the intensive care unit's electronic documentation system (Integrated Care Manager, Dräger Medical Deutschland GmbH, Lübeck, Germany).

This study was conducted according to the Declaration of Helsinki, local and institutional laws and was reported to the local ethical committee (No. WF-039/20). Written informed consent was waived for this kind of study.

### Treatment Protocol

All patients were treated according to common guidelines and as previously described (22). Intravenous nimodipine was administered when it was expected that the patient would remain sedated for a longer period of time, primarily in higher-grade SAH patients or patients suffering severe complications like rebleeding, or when oral medication was not safely absorbed enterally due to nausea and vomiting. After initiation of i.v. nimodipine (Nimotop S, Bayer Vital, Leverkusen, Germany) therapy with a continuous infusion rate of 2 mg/h, it was continued depending on the level of analgosedation or already evident vasospasms or perfusion deficits. The switch to enteral administration was then made with 60 mg orally 4 h apart. In patients unable to swallow but expected enteral absorption, tablets were crushed and washed down a nasogastric tube with normal saline (23, 29).

If vasospasm, perfusion deficits of one hemisphere or territorial, or neurological deficits were detected, and vasospasm was confirmed via digital subtraction angiography (DSA), i.a. spasmolysis with nimodipine was applicated via a microcatheter as rescue-therapy.

### Detection and Definition of Vasospasm and Delayed Cerebral Ischemia

Transcranial Doppler sonography of the anterior cerebral arteries (ACA) as well as the middle cerebral arteries (MCA) was performed daily beginning on the first day after admission via the transtemporal window. All measurements were performed with the same device (DWL Multi-Dop T Digital, Compumedics Germany GmbH, Singen, Germany) using a 2 MHz frequency ultrasound probe. The measurements were mainly carried out by the same trained medical technical assistant to avoid examiner-dependent bias. Mean flow velocities were recorded. Vasospasm was suspected by TCD if the mean flow velocity in the MCA was above 140 cm/s and above 120 cm/s in the ACA, respectively. In case of TCD suspicion of vasospasm we perform according to our local treatment protocol a computer tomography (CT) -scan, including computed tomography angiography (CTA) and CT-perfusion imaging (CTP). The occurrence of vasospasm and DCI was defined according to criteria published by Vergouwen et al. (30). In cases with proven high-grade vasospasm in CTA and/or a perfusion deficit in CTP a digital subtraction angiography (DSA) was then carried out (30).

### Definition of Subgroups

Depending on the neurological parameters and TCD, the following subgroups were defined to assess whether there were differences within these groups, which would further increase significance: (a) patients who had neither vasospasms nor infarctions, patients in whom either (b) vasospasms or (c) infarctions could be detected and (d) patients in whom both were present.

### Data Analysis

Medication was extracted as flowrate of a continuous application (nimodipine and norepinephrine) or as time of administration (nimodipine oral). Cumulative drug dosage over 24 h was calculated to perform possible correlations with measured TCD values (typically one per day). Quantitative variables were described as mean and standard deviation (SD), qualitative variables are outlined as number and percentage. The cumulative oral or i.v. dosage of nimodipine per day was used to characterize the phases of i.v. or oral administration of nimodipine. Between a dosage of 200 mg nimodipine orally and 10 mg i.v. per day administration was defined as oral. Values in between (≤ 200 mg/d orally and >10 mg/d nimodipine i.v.) were defined as overlap. I.v. administration was defined only if oral nimodipine was not administered. Only the switch from nimodipine i.v. to oral was considered.

Some patients had a longer overlapping time (max. 48 h) these values are depicted separately and not used for paired analysis. In some patients two phases of changes were presented, in these cases the last change was taken. We performed a gathered analysis of values from ACA and MCA and also of all values combined to show that even when combining values, no significant change was observed.

Statistical analysis of the data was performed by a univariate analysis using chi-squared tests, independent-samples Kruskal-Wallis tests or ANOVA tests depending on the scale of the measurements and equality of variances.

To examine correlations between the parameters we calculated the linear regression coefficient. The level of statistical significance was set at *p* < 0.05. To visualize parameters from the digital patient record, the time since ICU admission was analyzed by day and the mean (point) and standard error of the mean (error bars) were calculated. Data transformation, calculation and visualization was done in R (version 3.6.3 main packages: dplyr, tidyverse, stringr, ggplot2, ggpubr). For final composition of Figures Adobe Illustrator was used (Adobe Inc., San José, USA; Version 24.3).

## Results

A total of 133 patients initially received nimodipine i.v. after aneurysmal SAH, which was subsequently switched to oral administration after a mean of 11.7 ± 5.78 days. Forty six male and 87 female patients with a mean of 56 ± 13.7 years of age were included in this study. The aneurysms most frequently causing a SAH were situated in the anterior circulation (70.7%). Vasospasms were detected via CTA, CTP and DSA imaging in 30% of cases. Besides the detection of vasospasms 20.3 % of patients showed an infarction on imaging. In 14.3% of patients an infarction could be documented without any prior detection of vasospasm. 42.9% of the patients were classified as uncomplicated course of SAH since neither vasospasms nor infarctions occurred.

Patient cohorts were further divided and defined according to these findings: Patients who had an uncomplicated course (UC) represented the major group with 57 patients. CVS occurred in 30 cases (22.6%), 19 patients (14.3%) suffered from cerebral infarction without CVS (CI), and CVS+CI occurred in 27 cases (20.3%), Table 1.

**Table 1:** Patient characteristics.

	**UC**	**CVS**	**CI**	**CVS+CI**	***p*-value**
Number of patients (%)	57 (42.9)	30 (22.6)	19 (14.3)	27 (20.3)	
Sex—no. (%)					0.30
Male	22 (38.6)	7 (23.3)	9 (47.4)	8 (29.6)	
Female	35 (61.4)	23 (76.7)	10 (52.6)	19 (70.4)	
Age—mean (sd)	56.5 (14)	54.1 (13.4)	59.2 (13.4)	55.1 (13.9)	0.62
Localization of aneurysm (Circulation)—no. (%)					0.02
Anterior	39 (68.4)	20 (66.7)	10 (52.6)	25 (92.6)	
Posterior	18 (31.6)	10 (33.3)	9 (47.4)	2 (7.4)	
Hunt & Hess grade—no. (%)					0.79
1	12 (21.1)	4 (13.3)	5 (26.3)	5 (18.5)	
2	18 (31.6)	11 (36.7)	4 (21.1)	6 (22.2)	
3	9 (15.8)	8 (26.7)	4 (21.1)	6 (22.2)	
4	6 (10.5)	5 (16.7)	1 (5.3)	3 (11.1)	
5	12 (21.1)	2 (6.7)	5 (26.3)	7 (25.9)	
Fisher grade—median (IQR)	4.0 (±1.0)	4.0 (±1.0)	4.0 (±0.5)	4.0 (±0.5)	0.86
WFNS grade—median (IQR)	1.5 (±3.2)	2.0 (±2.8)	1.0 (±4.0)	2.5 (±3.8)	0.81
Initial GCS—median (IQR)	15.0 (±9.0)	14.0 (±3.5)	14.0 (±6.0)	13.0 (±8.5)	0.56
Pre-existing conditions—no. **(%)**					
Arterial hypertension	22 (38.6)	13 (43.3)	9 (47.4)	10 (37.0)	0.88
Alcohol abuse	5 (8.8)	1 (3.3)	0	0	0.20
Chronic headache	3 (5.3)	4 (13.3)	2 (10.5)	3 (11.1)	0.61
Diabetes mellitus	2 (3.5)	0	0	1 (3.7)	0.62
Cardiovascular disease	8 (14.0)	3 (10.0)	3 (15.8)	4 (14.8)	0.93
Smoking	13 (22.8)	6 (20.0)	5 (26.3)	6 (22.2)	0.97
Platelet aggregation inhibitors in premedication—no. (%)	32 (56.1)	11 (36.7)	12 (44.4)	16 (84.2)	0.13
Stay on ICU (days)—mean (sd)	17.5 (10.8)	19.2 (7.8)	24.3 (12.3)	18.7 (9.2)	0.05
Outcome—no. (%)					0.67
Deceased	9 (15.8)	3 (10)	6 (22.2)	3 (15.8)	
Survived	48 (84.2)	27 (90)	21 (77.8)	16 (84.2)	

*GCS, Glasgow Coma Scale; ICU, Intensive Care Unit; IQR, Interquartile Range; sd, Standard Deviation; WFNS, World Federation of Neurosurgical Societies*.

Mean flow velocities of the MCA 24 h before switching to oral administration were 83.82 cm/s (MCA) and 64.83 cm/s (ACA). On the first day after the switch mean flow velocities on TCD were 81.82 and 64.57 cm/s, respectively. This analysis was also performed depending on the occurrence of complications and examined for the above-mentioned subgroups.

There were no significant increases in mean flow velocities on TCD on the first day after switching the route of administration to oral administration in either subgroup of patients except for MCA in the CI group (Table 2). Mean flow velocities of the MCA in the UC group were 72.87 and 90.45 cm/s in the CVS+CI group. With respect to the ACA, mean values of 62.24 cm/s (UC) and 67.50 cm/s (CVS+CI) were recorded.

**Table 2:** Flow velocities values pre and post nimodipine switch.

**Subgroup**	**Pre in cm/sec** **mean (sd)**	**Post in (cm/sec)** **mean (sd)**	***p*-value**
**ACA**			
Overall population	64.83 ± 27.91	64.57 ± 22.75	0.93
UC	58.68 ± 22.26	62.24 ± 24.11	0.38
CVS	72.89 ± 34.43	66.00 ± 22.79	0.30
CI w/o CVS	60.40 ± 29.88	63.80 ± 19.14	0.45
CVS+CI	66.56 ±23.12	67.50 ± 23.31	0.90
**MCA**			
Overall population	83.82 ± 37.54	81.82 ± 30.53	0.48
UC	74.87 ± 31.73	72.87 ± 30.26	0.63
CVS	98.88 ± 46.19	92.97 ± 29.82	0.34
CI w/o CVS	62.36 ± 22.43	71.78 ± 22.26	<0.01
CVS+CI	93.80 ± 30.05	90.45 ± 30.36	0.67

*ACA, Anterior Cerebral Artery; CVS, Cerebral Vasospasm; CI, Cerebral Infarction; MCA, Middle Cerebral Artery; Pre = 1 day before nimodipine iv switch to oral administration, Post = first day of oral nimodipine administration, sd, Standard Deviation; UC, Uncomplicated Course; w/o, Without*.

**Figure 1:**
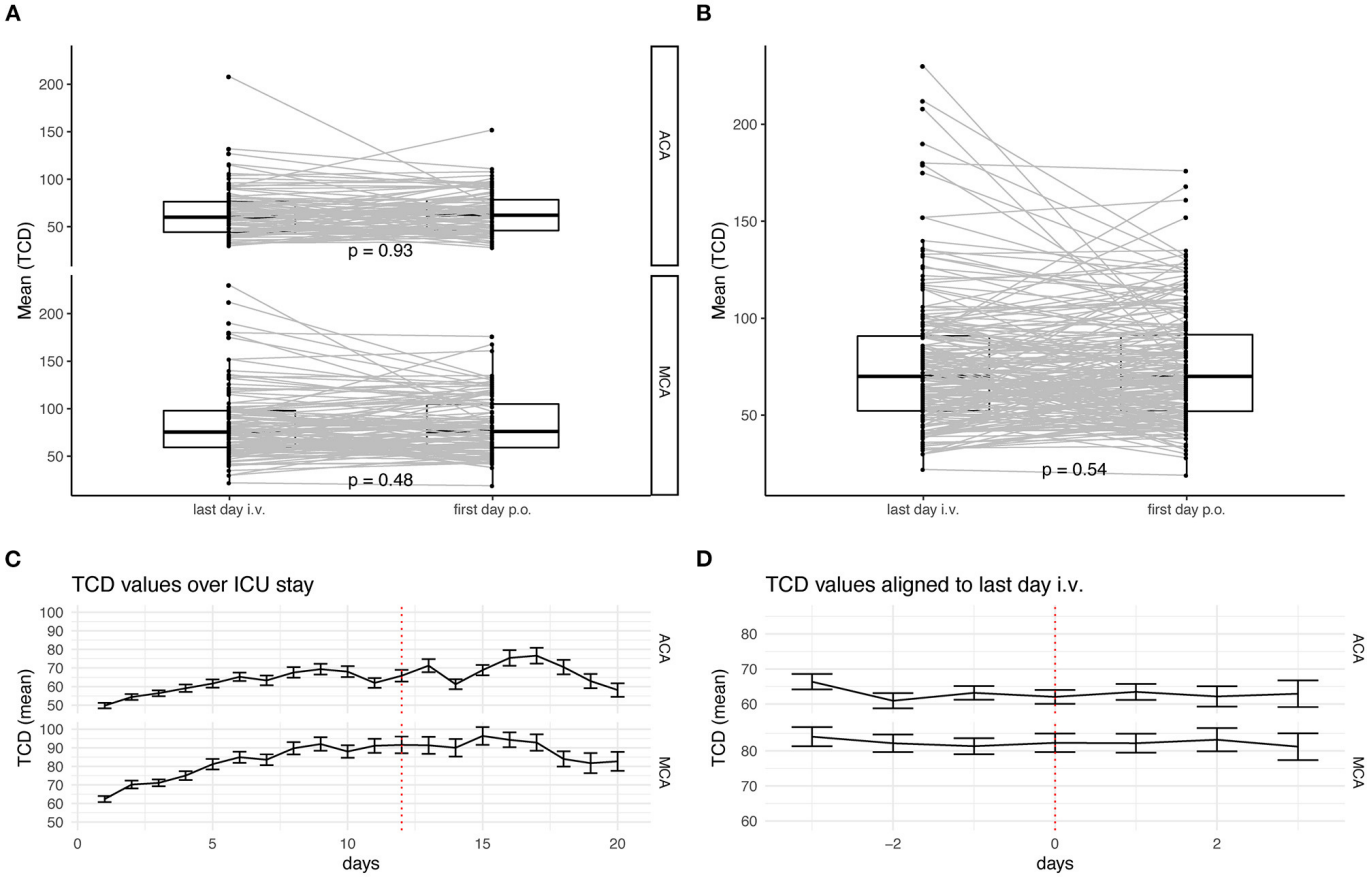
Analysis of TCD-values during the switch of the route of administration. **(A)** TCD values are measured longitudinal in patients. Only patients with consecutive values before and after switching the route of administration of nimodipine are depicted. Values for A. cerebri anterior (upper) and A. cerebri media (lower) are depicted separately. **(B)** All TCD values independent of their circulation are depicted. The lower and upper hinges of the boxplots correspond to the first and third quartile of all values. The whiskers represent the 1.5 inter quantile range (IQR). Dots show each value and grey lines connect individual patients' measurements. Statistical analysis was performed with paired *t*-test. **(C)** Mean TCD values are shown over the first 20 days of ICU stay for A. cerebri anterior (upper) and A. cerebri media (lower). Error bars represent the standard deviation. **(D)** Mean TCD values aligned to the individual switch of the route of administration. Whiskers represent. Error bars represent the standard deviation.

**Figure 2:**
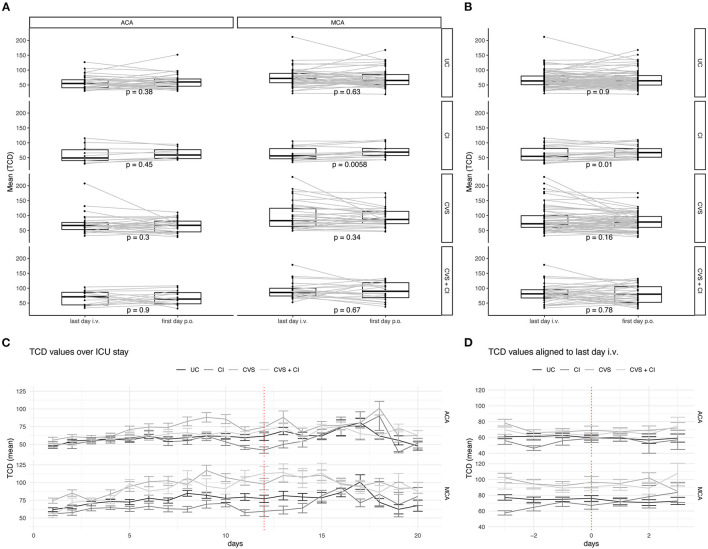
Analysis of TCD-values of subgroups during the switch of the route of administration. TCD values are measured longitudinal in all patients during the ICU stay. Grouping is done according to radiological findings: Uncomplicated Course (UC), Cerebral Infarction (CI), Cerebral Vasospasms (CVS), and Cerebral Vasospasm and Cerebral Infarctions (CVS + CI). **(A)** Only patients with consecutive values before and after switching the route of administration of nimodipine are depicted. Values for A. cerebri anterior (left) and A. cerebri media (right) are depicted separately. **(B)** All TCD values independent of their circulation are depicted. The lower and upper hinges of the boxplots correspond to the first and third quartile of all values. The whiskers represent the 1.5 inter quantile range (IQR). Dots show each value and grey lines connect individual patients' measurements. Statistical analysis was performed with paired *t*-test. **(C)** Mean TCD values are shown over the first 20 days of ICU stay for A. cerebri anterior (upper) and A. cerebri media (lower). Error bars represent the standard deviation. **(D)** Mean TCD values aligned to the individual switch of the route of administration. Whiskers represent. Error bars represent the standard deviation.

An analysis of the last day of i.v. administration of nimodipine vs. the first day of the full oral dose was performed. There were no significant changes in the flow velocities of the middle and anterior cerebral arteries (Figures 1A,B). The mean TCD values measured in the course of time during the intensive care stay and especially during the switch are shown in Figures 1C,D. For a more detailed description of the heterogeneous collective, the above-mentioned subdivision into subgroups was applied; in addition, the values for ACA and MCA are listed separately (Figure 2). There were no significant increases after switching the type of administration.

**Figure 3:**
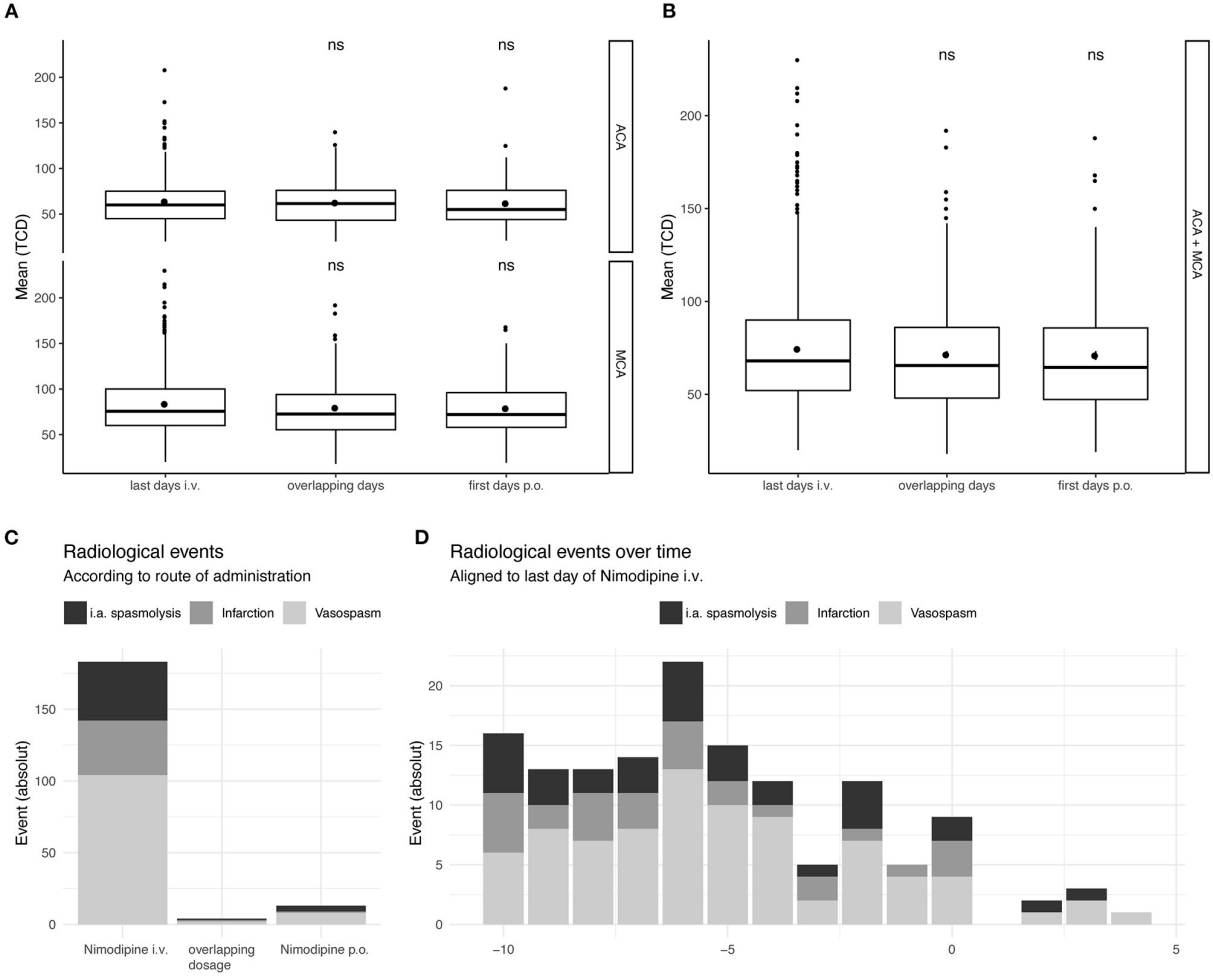
Temporal resolution of complications in subgroups in relation to the timing of the switch. Taking all measured TCD values in account of all patients included in the study not only longitudinal but all values were considered. TCD values on the last 2 days of Nimodipine iv. and the first 2 days of oral administration only were depicted, together with overlapping dosages. **(A)** Values for A. cerebri anterior (upper) and A. cerebri media (lower) are depicted separately. **(B)** All TCD values independent of their circulation are depicted. The lower and upper hinges of the boxplots correspond to the first and third quartile of all values. The whiskers represent the 1.5 inter quantile range (IQR). Statistical analysis was performed with unpaired *t*-test compared to the last day of i.v. administration. **(C)** Absolute amount of the radiological events are show according to the administered drug. **(D)** Radiological events over all the ICU stay (Absolute numbers). ns, not significant.

**Figure 4:**
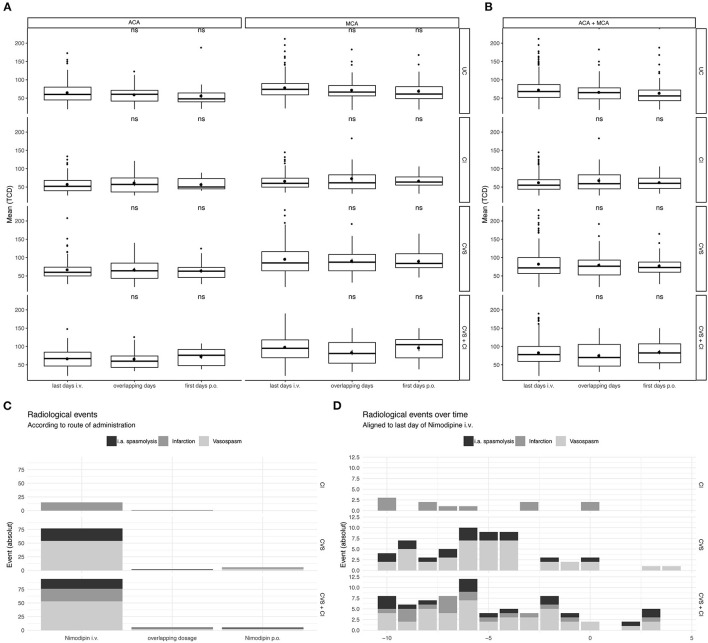
Temporal resolution of complications in subgroups in relation to the timing of the switch. Taking all measured TCD values in account of all patients included in the study not only longitudinal but all values were considered. TCD values on the last 2 days of Nimodipine iv. and the first 2 days of oral administration only were depicted, together with overlapping dosages. Grouping is done according to radiological findings: Uncomplicated Course (UC), Cerebral Infarction (CI), Cerebral Vasospasms (CVS), and Cerebral Vasospasm and Cerebral Infarctions (CVS + CI). **(A)** Values for A. cerebri anterior (upper) and A. cerebri media (lower) are depicted separately. **(B)** All TCD values independent of their circulation are depicted. The lower and upper hinges of the boxplots correspond to the first and third quartile of all values. The whiskers represent the 1.5 inter quantile range (IQR). Statistical analysis was performed with unpaired *t*-test compared to the last day of i.v. administration. **(C)** Absolute amount of the radiological events are show according to the administered drug. **(D)** Radiological events over all the ICU stay (Absolute numbers). ns, not significant.

No significant increase in flow velocities occurred during the switch or on the day after the switch compared to the day before the switch (last day i.v.) (Figures 3A,B). In none of the groups with complications (CVS, CI and CVS+CI) was there a significant increase in flow velocities during the switch or on the days after the switch compared to the days before the switch (last day i.v.) (Figures 4A,B). In an unpaired analysis, the combination of flow velocities of ACA and MCA showed significantly lower flow velocities among the uncomplicated courses on the first day after the switch (Figure 3B). No further significant changes on TCD during the switching period were found (Figures 3A,B).

In a next step, we examined the time course of the complications that occurred in relation to the timing of oralization. There was no accumulation of complications (CVS, CI) after or during the switch of the route of administration (Figures 3C,D, 4C,D): During the intravenous administration phase, 93 events occurred in the CVS+CI group. Of these, 18 were intraarterial spasmolyses with locally administered nimodipine. After the switch, 8 events occurred in the same group, of which 3 were intraarterial spasmolyses with locally administered nimodipine.

Cerebral infarctions were mainly detected in the first 10 days of ICU stay, which is in line with detection of CVS. Within the first 10 days, 37 of a total of 46 cerebral infarctions occurred. Furthermore, 93 of a total of 124 cerebral vasospasms occurred and 35 of a total of 50 i.a. spasmolyses were performed within the first 10 days.

Systemic mean arterial pressure remained stable during the switch (not shown). Furthermore, the mean catecholamine dose decreased significantly in all groups after the switch. Before the switch the dose ranged from 7 to 13 mg norepinephrine/day (depending on the group, Table 3) compared to 4–8 mg/day after the switch.

**Table 3:** Norepinephrine dose around nimodipine switch [mg/day].

	**Pre mean (sd)**	**Post mean (sd)**	***p*-value**
UC	9 (7)	4 (5)	<0.0001
CI	7 (7)	4 (3)	0.03
CVS+CI	13 (10)	8 (6)	<0.0001

**Figure 5:**
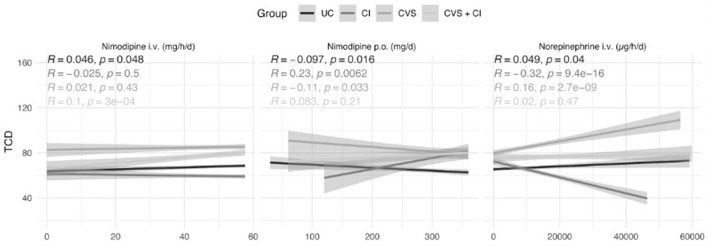
Correlation of Transcranial Doppler Sonography to oral and i.v. nimodipine. Groups: Red line—uncomplicated; green line Infarcts, blue line vasospasms, and purple line vasospasms and infarcts. To unveil dosage effects correlation analysis were done. All TCD values are depicted and correlated to the Nimodipine iv., oral and Norepinephrine cumulative dosage over 24 h. Linear regression line with the according standard error are depicted for the defined groups. Pearson correlation coefficient and the according *p*-value are shown in the upper left of the plots.

To further address the changes of nimodipine dosage and their influence to the underlying changes in flow velocities correlation analyses were performed (Figure 5).

While i.v. nimodipine shows no relevant correlation, oral nimodipine shows a negative correlation coefficient in the UC- and CVS group.

## Discussion

The evidence for oral administration of nimodipine is derived from randomized-controlled trials. However, both intravenous and oral administration of nimodipine are common practice and, in addition to intra-arterial rescue therapy, have a relevant role in everyday clinical practice (31). Some studies showed no significant difference in terms of plasma concentration or efficacy achieved when comparing oral with intravenous administration (32–34). The need to administer norepinephrine, which often accompanies intravenous administration, initially keeps patients in the ICU. Transfer to a peripheral ward is therefore often only possible after oral administration and thus absence of catecholamines. Hence the desire for a rapid switch to oral administration. Despite the known data with regard to bioavailability, to the best of our knowledge, studies investigating parameters at the point of switch from intravenous to oral nimodipine administration are completely missing in the literature (22, 23).

The discussed lower oral bioavailability of nimodipine, especially in analgosedated patients, as well as the frequent blood pressure-related interruption of oral administration make i.v. administration of the drug a valid and common alternative (23–25). Sandow et al. were able to demonstrate that low blood pressure often leads to dose reductions or interruption of oral dosing, which may be associated with unfavorable outcome (25). When administered intravenously, there is a reduced risk of lower bioavailability in sedated patients. Intravenous nimodipine therapy is therefore often used initially in the intensive care therapy of SAH patients followed by a switch to oral administration in the later, more stabilized phase.

In the context of this clinically relevant topic, we were able to show that almost no significant change in flow velocities or incidence of negative clinical events occurred after the switch. Although flow velocities in the CI group increase significantly on the first day after the switch (Table 2), this is apparently clinically not relevant as there seems to be no clustering of adverse events (Figure 4D).

Since our patient cohort is heterogeneous including patients with complications as well as uncomplicated courses, all parameters in the subgroups in which complications occurred were systematically examined to increase the sensitivity of the analyses.

Even though we explicitly looked into groups with complications and compared them with uncomplicated courses, we found no differences.

It should be noted that the switch was performed at a mean of 12 days after admission to the ICU and thus at a time when the risk for vasospasm is still present but decreasing (35, 36).

This study has a number of limitations, particularly its retrospective, single-center design. Despite the relatively small sample size, demographics correspond well with other SAH cohorts and epidemiological data.

The potential benefit of initial intravenous nimodipine therapy is still unclear and will have to be investigated in prospective studies. Our data and the considerations for intravenous administration may be relevant for studies comparing standard nimodipine therapy with an investigational drug like the NEWTON trial (37).

In conclusion, we found no indication of safety concerns when switching from initial intravenous nimodipine administration in the acute phase to subsequent oral administration. The switch was neither associated with clinically relevant increases in TCD-velocities nor with other relevant adverse events.

## Data Availability Statement

The raw data supporting the conclusions of this article will be made available by the authors, without undue reservation.

## Ethics Statement

The studies involving human participants were reviewed and approved by Hamburg Ethical Committee Weidestr. 122 b 22083 Hamburg. Written informed consent for participation was not required for this study in accordance with the national legislation and the institutional requirements.

## Author Contributions

JG supervised the study, designed the study question, interpreted the data, and drafted the manuscript. NS conceived the study question and analyzed and interpreted the data. JCG collected the data. CG contributed to the data interpretation. MW helped to interpret the data and conceive the study questions. PC contributed to the overall design of the study, supervised the study, conceived the study question, designed the analysis plan, and analyzed and interpreted the data. All authors revised the manuscript.

## Conflict of Interest

The authors declare that the research was conducted in the absence of any commercial or financial relationships that could be construed as a potential conflict of interest.

## Publisher's Note

All claims expressed in this article are solely those of the authors and do not necessarily represent those of their affiliated organizations, or those of the publisher, the editors and the reviewers. Any product that may be evaluated in this article, or claim that may be made by its manufacturer, is not guaranteed or endorsed by the publisher.
