# Supplementary material for: Safety and Clinical Effects of Switching From Intravenous to Oral Nimodipine Administration in Aneurysmal Subarachnoid Hemorrhage
Source: Front Neurol. 2021 Nov 16;12:748413. doi: 10.3389/fneur.2021.748413 (PMC8636241; doi:10.3389/fneur.2021.748413)

Supplemental Figure 1

Flow diagram of patients included in this study and inclusion and exclusion criteria

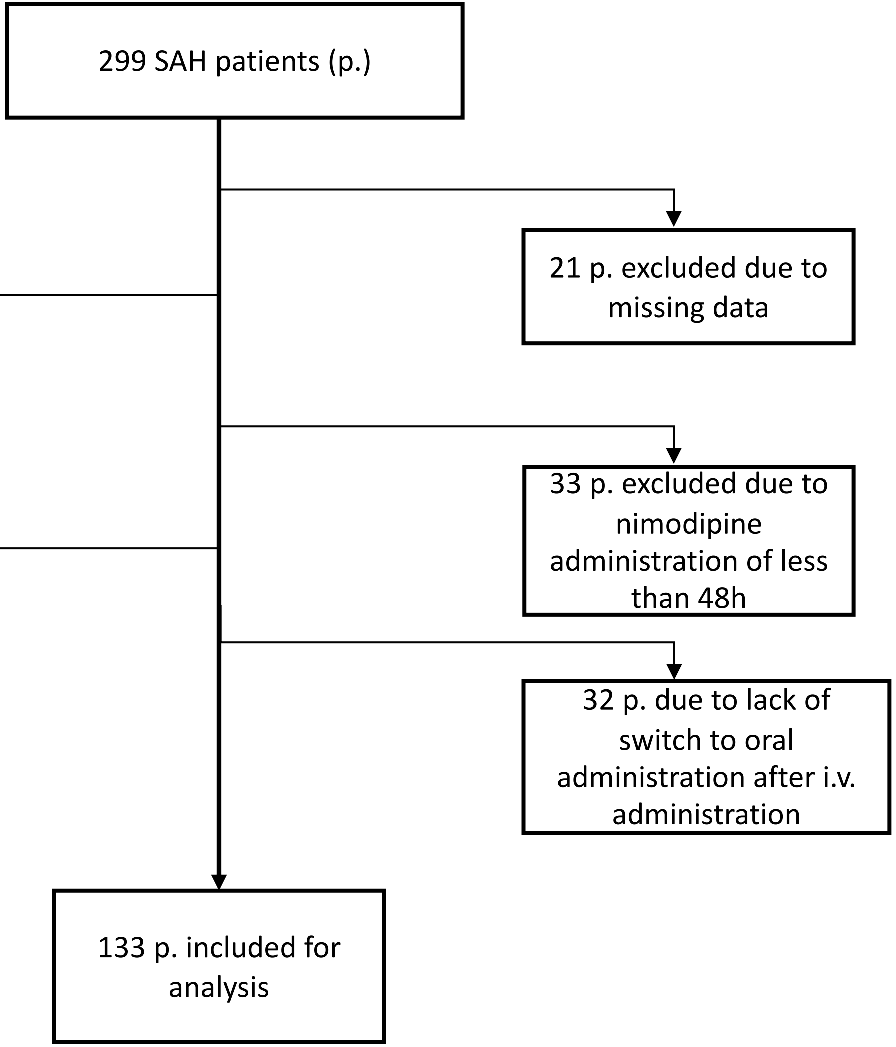

Supplement: Supplementary file 1 [file Image_1.PDF]
